# What does body size mean, from the “plant's eye view”?

**DOI:** 10.1002/ece3.2476

**Published:** 2016-09-22

**Authors:** Amanda J. Tracey, Kimberly A. Stephens, Brandon S. Schamp, Lonnie W. Aarssen

**Affiliations:** ^1^ Department of Biology Queen's University Kingston ON Canada; ^2^ Department of Biology Algoma University Sault Ste. Marie ON Canada

**Keywords:** body mass density, competition, dry mass, fitness, fresh mass, plant size, reproductive economy, space occupancy body size

## Abstract

Alternative metrics exist for representing variation in plant body size, but the vast majority of previous research for herbaceous plants has focused on dry mass. Dry mass provides a reasonably accurate and easily measured estimate for comparing relative capacity to convert solar energy into stored carbon. However, from a “plant's eye view”, its experience of its local biotic environment of immediate neighbors (especially when crowded) may be more accurately represented by measures of “space occupancy” (S–O) recorded *in situ—*rather than dry mass measured after storage in a drying oven. This study investigated relationships between dry mass and alternative metrics of S–O body size for resident plants sampled from natural populations of herbaceous species found in Eastern Ontario. Plant height, maximum lateral canopy extent, and estimated canopy area and volume were recorded *in situ* (in the field)—and both fresh and dry mass were recorded in the laboratory—for 138 species ranging widely in body size and for 20 plants ranging widely in body size within each of 10 focal species. Dry mass and fresh mass were highly correlated (*r* ^2^ > .95) and isometric, suggesting that for some studies, between‐species (or between‐plant) variation in water content may be unimportant and fresh mass can therefore substitute for dry mass. However, several relationships between dry mass and other S–O body size metrics showed allometry—that is, plants with smaller S–O body size had disproportionately less dry mass. In other words, they have higher “body mass density” (BMD) — more dry mass per unit S–O body size. These results have practical importance for experimental design and methodology as well as implications for the interpretation of “reproductive economy”—the capacity to produce offspring at small body sizes—because fecundity and dry mass (produced in the same growing season) typically have a positive, isometric relationship. Accordingly, the allometry between dry mass and S–O body size reported here suggests that plants with smaller S–O body size—because of higher BMD—may produce fewer offspring, but less than proportionately so; in other words, they may produce more offspring per unit of body size space occupancy.

## 1 Introduction

Competition is one of the most important processes affecting the structure of plant communities (Tilman, 1982). Many previous experimental studies have confirmed that smaller species routinely experience more suppressed growth than larger species when they compete (Gaudet & Keddy, 1988; Goldberg & Landa, 1991; Jumpponen, Nulder, Huss‐Daniels, & Högberg, 2005; Keddy, 2001; Keddy, Nielsen, Weiher, & Lawson, 2002; Keddy & Shipley, 1989; Keddy, Strutt, & Wisheu, 1994; Rosch, Van Rooyen, & Theron, 1997; Violle et al., 2009; Wang, Stieglitz, Zhou, & Cahill, 2010). Therefore, according to traditional theory, superior competitive ability in plants requires the capacity for a relatively large plant body size (Goldberg, 1996; Grace, 1990; Grime, 1973), and the vast majority of previous studies have quantified variation in plant competitive ability in terms of measures that reflect ability to impose growth suppression on neighbors (Aarssen & Keogh, 2002).

This “size advantage,” however, is not reflected in plant body size distributions which show that most species are actually relatively small, at virtually all scales (Aarssen, Schamp, & Pither, 2006; Dombroskie & Aarssen, 2010; Tracey & Aarssen, 2011). Being able to grow to a large body size will be adaptive under competition (in terms of reproductive success), but only if large body size can be attained. Importantly, this is almost never the case for the resident plants within a natural population; the vast majority are usually severely crowded and remain as suppressed weaklings until death (Chambers & Aarssen, 2009; Tracey & Aarssen, 2014). Greater success in competition therefore may not usually involve a size advantage at all, but rather a “reproductive economy advantage” (Aarssen, 2008, 2015; Aarssen et al., 2006), that is, capacity to produce at least some offspring despite severe size suppression due to crowding. Few studies to date have measured competitive ability in terms of reproductive success (Neytcheva & Aarssen, 2008).

Body size in plants has been measured most commonly in terms of height, stem diameter, and dry mass. Relationships between these metrics have been studied both between‐ and within‐species (Anten & Hirose, 1999; Henry & Aarssen, 1999; Mandak & Pysek, 1999; Nagashima & Terashima, 1995; Niklas, 1995; Reddy, Pachepsk, & Whisler, 1998; Weiner & Fishman, 1994; Weiner & Thomas, 1992). In many cases, they are allometric, that is, as height increases, dry mass increases disproportionately, but this can depend on variation in the severity of crowding or the relative intensity of intraspecific versus interspecific competition (Weiner, 1990). In some cases, when crowding increases, plants may increase resource allocation to emphasize vertical growth more than stem diameter or mass, thus reducing the likelihood of being outcompeted for sunlight (Nagashima & Terashima, 1995; Weiner & Fishman, 1994; Weiner & Thomas, 1992). This allometry may also be genetically fixed; species‐specific allometry for height versus stem diameter, for example, has been commonly reported for trees (Weller, 1987; White, 1981).

Because of their allometric relationships, plant height, stem diameter, and dry mass will not be generally interchangeable as metrics of body size without altering interpretations of the causes and effects of body size variation. Consequently, the vast majority of research in herbaceous plants has used only dry mass to measure variation in plant body size. This represents a reasonably accurate estimate for comparing the relative capacity of plants to capture and convert solar energy into stored carbon. But dry mass is also commonly favored (rather than fresh mass) because it allows control for effects of variation in water content (between‐species, and between individual plants) resulting from uncontrolled spatial and temporal environmental variation in water availability.

Water, however, is one of the main resources that plants compete for. Removing it from measurement thus removes information about potential variation (between‐species or between‐plants) in water uptake success—hence, ironically, it removes information about variation in abilities to deny water to neighbors. More generally, when a plant competes for resources *in situ*, it does so with its fresh mass, and its impact on neighbors will be a function of the three‐dimensional space that its fresh mass occupies (Schamp & Aarssen, 2014). John Harper (1977) famously suggested that “… the ‘plant's‐eye view’ is what is relevant to explain the distribution, adaptation and the process of change within species and within communities” (p. 706). This view emphasizes the experience and consequences of near‐neighbor interactions within local neighborhoods (Aarssen, 1989). Accordingly, the effects of, and responses to, variation in neighbor effects, we predict, should be understood more accurately by measuring fresh mass of resident plants and their success in terms of space occupancy *in situ* than by measuring dry mass after storage in a drying oven.

We studied 138 herbaceous species to explore the implications of body size variation from the “plant's eye view”—that is, with body size measured not just as dry mass, but also in terms that more directly reflect what the plant experiences, and the space that it occupies, within its local neighborhood: its fresh mass, height, and maximum lateral canopy extent, area, and volume. Specifically, we tested whether relationships between dry mass and “space occupancy” (S–O) body size metrics are proportional (isometric vs. allometric), and we assessed the implications of proportionality (or departure from it) when using relative body size to predict reproductive success, and consequent success in gene transmission, when body size is limited by neighborhood crowding.

## 2 Materials and methods

### 2.1 Study sites and study species

Sampling was conducted as time permitted between May and October 2013 from natural plant populations found in a variety of habitats (old‐field meadows, fence rows, and roadside edges) in southern Ontario, mainly in the vicinity of Kingston, Ontario, Canada (44°15′N, 76°30′W), including Queen's University Biological Station (44°33′N, 76°21′W). Candidate species were chosen to include a wide range of species body sizes (based on visual estimation).

### 2.2 Field sampling and data collection

Populations were sampled when resident plants were at the reproduction stage to ensure that body size was at or approaching maximum for the current growing season. Only populations with a minimum of 20 resident plants were used, and where possible, up to five populations at least 2 km apart were sampled for each species. For each population, the largest individual “rooted unit” (Aarssen, 2014) of each species (based on visual estimation) was chosen for sampling. Individuals showing signs of heavy herbivore damage (e.g., missing stems indicated by breakage points) were avoided. In a few cases, where only one population was located, the five largest individuals were collected from that population.

Ten candidate species with population sizes >100 were also chosen for within‐species analyses. In each case, the largest and smallest resident reproductive plants were sampled as well as 18 additional reproductive plants chosen haphazardly to represent the range of resident body sizes between the largest and smallest (20 per species).

For each sampled individual, height, maximum lateral canopy extent, and the perpendicular lateral canopy extent (at the widest point along the maximum lateral extent) were measured. The above‐ground portion of each individual was then harvested, and fresh mass was recorded in the laboratory using an analytical balance. Each individual was then placed in a drying oven at 80°C for 72 hr and then reweighed to obtain dry mass.

### 2.3 Data analyses

Mass‐based body size for the sampled individuals was represented by above‐ground fresh mass and dry mass. “Space occupancy” body size involved four metrics: plant height, maximum lateral canopy extent, estimated canopy area (the product of maximum lateral canopy extent and the perpendicular lateral canopy extent), and estimated canopy volume (the product of height and estimated canopy area). Fresh mass, dry mass, height, and maximum lateral canopy extent are absolute measures of plant body size, whereas the estimates of canopy area and volume represent relative measures of plant body size. For the between‐species analysis, only the individual with the largest dry mass was used.

To account for possible effects of phylogeny on between‐species trait relationships, phylogenetic trees (Fig. S1) were constructed using Phylomatic (version 3.0, http://www.phylodiversity.net, Webb et al. 2011) and using the rooted vascular plant megatree from Zanne et al. (2014). Standardized PI (phylogenetically independent) contrasts were computed for log‐transformed traits using R (R Development Core Team 2012), the ape package version 3.4 (Paradis et al. 2004), and the phytools package (Revell, 2012).

Standard tests for proportionality (iso/allometric) relationships between species trait PI contrasts were carried out using Type I linear regression (with SigmaPlot 10 (2006) and Type II (reduced major axis; RMA) scaling coefficients were calculated based on Zar (2002). [RMA regression recognizes error in measurement of both the x‐ and *y*‐axis variables, and combined with log‐transformation allows assessment of proportionality without assumption about which variable is dependent on the other]. All data were log‐transformed to obtain normal distributions and to enable the use of slopes for evaluating isometry before contrasts were applied. For each regression, the null hypothesis is that body size metrics are isometric; that is, an increase in one metric is accompanied by a proportional increase in the other. Two‐tailed *t*‐tests were used for detecting departure of RMA regression slopes from isometry (i.e., to test for allometry). Height and maximum lateral canopy extent scale in one dimension, canopy area scales in two dimensions, and canopy volume and mass scale in three dimensions. Accordingly, both mass–height and mass–maximum lateral canopy extent relationships have isometric slopes of 3/1 (or 3), mass–canopy area relationships have an isometric slope of 3/2, and mass‐canopy volume relationships have an isometric slope of 3/3 (or 1).

## 3 Results

### 3.1 Between‐species relationships

Data collection involved a total of 138 species (Table S1) with mass values spanning four orders of magnitude. The data set was heavily biased by Asteraceae, comprising almost 1/3 (n = 41) of the collected species, which were thus analyzed separately so as not to dominate the phylogenetically independent contrast analysis (Fig. S1).

**Figure 1:**
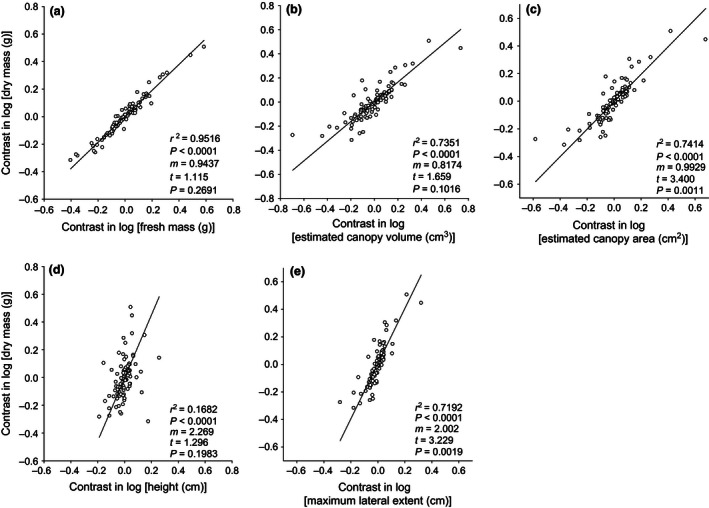
Between‐species relationships (n = 97 species based on PI contrasts in log‐transformed data) for above‐ground dry mass verses each of five other metrics of body size recorded for individuals sampled from natural populations, not including those from the Asteraceae family. For each species, the individual with the largest above‐ground dry mass is paired with its fresh mass (a), estimated canopy volume (b), estimated canopy area (c), height (d), and maximum lateral canopy extent (e). *r* ^2^ and associated *p*‐values are from Type I linear regression analysis. Solid lines are from RMA regression analyses; m = RMA slope; *t* and associated *p*‐values test for deviation from the null hypothesis of isometry—which is 1:1 in (a) and (b), where both metrics scale in three dimensions; 3:2 in (c), where one metric scales in three dimensions and the other scales in two dimensions; and 3:1 in (d) and (e), where one metric scales in three dimensions and the other scales in one dimension

**Figure 2:**
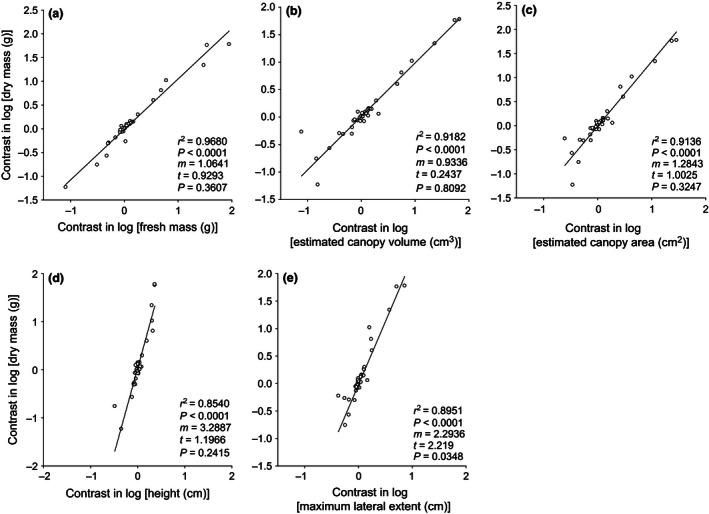
Between‐species relationships (n = 41 species based on PI contrasts in log‐transformed data) for above‐ground dry mass verses each of five other metrics of body size recorded for individuals sampled from natural populations, including only those species from the Asteraceae family. For each species, the individual with the largest above‐ground dry mass is paired with its fresh mass (a), estimated canopy volume (b), estimated canopy area (c), height (d), and maximum lateral canopy extent (e). *r* ^2^ and associated *p*‐values are from Type I linear regression analysis. Solid lines are from RMA regression analyses; m = RMA slope; *t* and associated *p*‐values test for deviation from the null hypothesis of isometry—which is 1:1 in (a) and (b), where both metrics scale in three dimensions; 3:2 in (c), where one metric scales in three dimensions and the other scales in two dimensions; and 3:1 in (d) and (e), where one metric scales in three dimensions and the other scales in one dimension

Dry mass and fresh mass had an isometric relationship (i.e., slope not significantly different from 1.0) (Figures 1a, 2a). The slope for dry mass versus height was also isometric (i.e., not significantly different from 3) (Figures 1d, 2d). Dry mass versus maximum lateral canopy extent was significantly allometric (with slope significantly <3) for both the multifamily data set (*t* = 3.229, *p* = .0019; Figure 1e) and the Asteraceae (*t* = 2.219, *p* = .0348; Figure 2e). Dry mass versus estimated canopy area was significantly allometric (with slope significantly <1.5) for the multifamily data set (*t* = 3.40, *p* = .0011; Figure 1c) but not for the Asteraceae (Figure 2c). For dry mass versus estimated canopy volume, the slope was numerically less than the expected isometric slope of 1.0 for the multifamily data set, and this allometry was marginally significant (*t* = 1.659, *p* = .10; Figure 1b)—but this relationship was not significantly different from isometry for the Asteraceae (Figure 2b). Similar relationships were found using raw data (log‐transformed) as opposed to contrasts (Fig. S7).

### 3.2 Within‐species relationships

Within species, dry mass and fresh mass were isometrically related (slope not significantly different from 1.0) for all 10 study species (Table 1; Figs S2–S6, panels a and f). However, the balance of relationships between dry mass and space occupancy body size metrics was allometric (Table 1). For dry mass versus canopy volume, relationships were numerically less than isometric for all 10 species and this allometry was at least marginally significant (*p* < .10) for *Erigeron strigosus* (*p* = .0801; Fig. S2b), *Dianthus armeria* (*p* = .0052; Fig. S3b), *Matricaria discoidea* (*p* = .0644; Fig. S4g), and *Barbarea vulgaris* (*p* = .0428; Fig. S6g). Slopes of the dry mass–canopy area relationship were all numerically less than expected under isometry; allometry was significant for *Dianthus armeria* (*p* = .0033; Fig. S3c) and *Barbarea vulgaris* (*p* = .0092; Fig. S6h). Similarly, for dry mass versus maximum lateral canopy extent, relationships were again numerically less than isometric for all study species and this allometry was significant for *Dianthus armeria* (*p* = .0071; Fig. S3e), *Oxalis corniculata* (*p* = .0542; Fig. S6e), and *Barbarea vulgaris* (*p* = .0317; Fig. S6j). Finally, for dry mass versus height, slopes for some species were numerically less than isometric, and significantly so for *Erigeron strigosus* (*p* = .0925; Fig. S2d) and *Matricaria discoidea* (*p* = .0466; Fig. S4i)—while for other species, slopes were numerically greater than isometric, and significantly so for *Hesperis matronalis* (*p* = .0275; Fig. S2i) and *Rumex acetosella* (*p* = .0865; Fig. S5d).

**Table 1:** Summary of within‐species (n = 20) relationships for dry mass versus each of five other metrics of body size for 10 selected species sampled from natural populations

Species	Fresh mass (H_0_: slope = 1)		Estimated canopy volume (H_0_: slope = 1)		Estimated canopy area (H_0_: slope = 1.5)		Height (H_0_: slope = 3)		Maximum lateral canopy extent (H_0_: slope = 3)	
	Slope	*p*‐value	Slope	*p*‐value	Slope	*p*‐value	Slope	*p*‐value	Slope	*p*‐value
*Barbarea vulgaris*	1.0023	.9624	0.7457	**.0428**	0.9475	**.0092**	3.3125	.5766	1.8915	**.0317**
*Dianthus armeria*	0.9916	.9128	0.4890	**.0052**	0.6149	**.0033**	2.0852	.129	1.3957	**.0071**
*Echium vulgare*	0.8884	.1038	0.9795	.8633	1.2716	.1551	3.9763	.2511	2.5332	.1945
*Erigeron strigosus*	1.0104	.755	0.6754	**.0801**	0.9414	.103	2.1037	**.0925**	1.7368	.1029
*Hesperis matronalis*	1.0026	.9361	0.9309	.653	1.1046	.1276	5.0769	**.0275**	2.1176	.1957
*Matricaria discoidea*	1.0104	.8846	0.7215	**.0644**	1.1327	.1004	1.9408	**.0466**	2.5355	.3601
*Oxalis corniculata*	0.9341	.209	0.8520	.4552	1.1062	.2000	3.0676	.9457	1.8307	**.0542**
*Plantago lanceolata*	0.9604	.5328	0.8864	.589	1.1524	.2372	2.8744	.916	2.5674	.5411
*Rumex acetosella*	1.0512	.2431	0.9554	.8392	1.1462	.2758	4.9951	**.0865**	2.1860	.3253
*Verbascum thapus*	1.0465	.3471	0.9777	.8658	1.3603	.4844	3.4062	.4378	2.9068	.8153

Slopes are from RMA regression analyses, and *p*‐values are from *t*‐tests for deviation from the null hypothesis (H_0_) of isometry. *p* ‐values <.01 are bolded. Dry mass scales in three dimensions, and so hypothetical isometric relationships are represented by: a slope of 1 for regressions with fresh mass and with estimated canopy volume, which also both scale in three dimensions; a slope of 3/2 (1.5) for regressions with estimated canopy area, which scales in two dimensions; and a slope of 3 for regressions with height and maximum lateral canopy extent, which both scale in only one dimension.

## 4 Discussion

Most studies of competitive ability in herbaceous plants measure performance in terms of dry mass, despite the fact that between‐plant variation in water content may be at least in part a consequence of differential success in denying water to neighbors when water is a contested resource. The important question then is as follows: To what extent are fresh and dry mass correlated? Schamp and Aarssen (2014) found, in a greenhouse study, that a 10‐fold variation in fresh mass across 10 study species was uncorrelated with dry mass. In our data for field collected plants, however, the between‐species relationships for above‐ground fresh mass and dry mass (spanning about four orders of magnitude) were highly correlated and isometric (Figures 1a, 2a). The same was true for the between‐plant relationship (n = 20) for each of 10 species with mass values spanning one to three orders of magnitude (Figs S1–S5). What's more, these fresh mass–dry mass relationships are extremely strong (all *r* ^2^ > .95) given the potential for different water conditions across populations and harvest times. These results suggest that dry mass and fresh mass are generally interchangeable when examining between‐species body size variation in plants, at least for the species examined here, and this could help inform researchers when making important decisions regarding experimental design and methodology. The difference between field and experiment here may derive from the more complex interaction between water and the availability of soil nutrients in field conditions. It remains to be seen whether fresh and dry mass relationships vary across gradients of moisture and other soil nutrients in natural conditions.

Among species, dry mass and height were isometrically related (Figure 1d, 2d), while this relationship within species was variable, with some species having slopes significantly greater than, or less than, isometric (Table 1, Figs S1–S5). These variable results are consistent with what has been observed for the height–dry mass relationship in other studies (Anten & Hirose, 1999; Mandak & Pysek, 1999; Nagashima & Terashima, 1995; Weiner & Thomas, 1992). However, it is difficult to compare our results with these studies as it is not clear whether Type II regressions were used, which can compromise interpretation (Henry & Aarssen, 1999).

**Figure 3:**
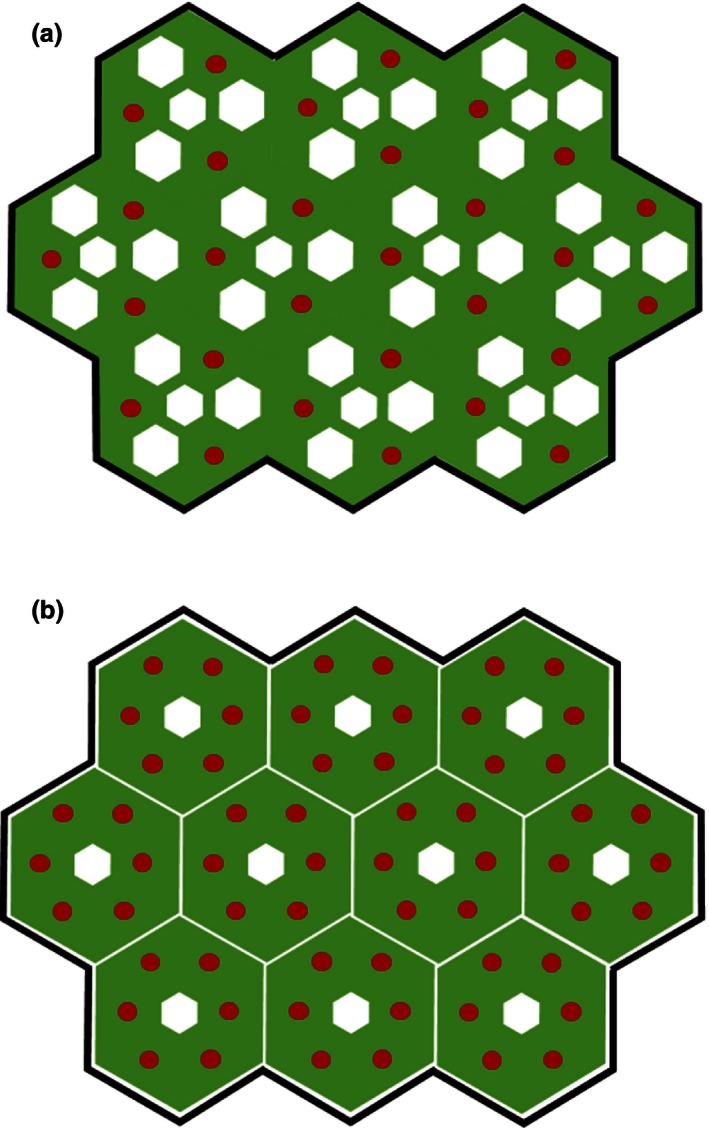
Symbolic representation of the effect of individual plant body size (i.e., size of an individual “rooted unit”) on “body mass density” (above‐ground dry mass per unit space occupancy). Panel (a) represents a single adult offspring individual produced by a parent plant A belonging to a species with a large maximum potential body size (MAX), and panel (b) shows 10 individual adult offspring produced by a parent plant B belonging to a species with a much smaller MAX. Panels (a) and (b) could also represent different individual plant sizes from the same species, for example, growing under uncrowded versus crowded conditions, respectively. In this scenario, the one large plant in (a) has the same above‐ground “space occupancy” as the 10 small plants in (b), that is, the same total canopy extent in terms of area or volume represented by the black perimeter. The relative mass per plant is represented by the amount of green space, mass gaps in the plant's canopy are represented by white space, and seed production is represented by red circles

We found that dry mass per unit of canopy volume, canopy area, and maximum lateral canopy extent (measures of S–O body size) was generally greater for smaller species than for larger species (Figures 1b,c,e, 2008e) and also for smaller plants than for larger plants within species (Table 1, Figs S1–S5). A relatively large body size is commonly associated with biomechanical and/or physical constraints (Givnish, 1986; McMahon, 1975). In other words, larger species (and larger plants within species) normally distribute their above‐ground dry mass more sparsely within their occupied space (Schamp & Aarssen, 2010). Here, we refer to this as a lower “body mass density” (BMD), that is, dry mass per unit space occupancy. This is illustrated in Figure 3, where a single adult offspring (an individual “rooted unit”; Aarssen, 2014) produced by a parent plant belonging to a species with a large maximum potential body size (MAX) (Figure 3a) is depicted as having the same above‐ground space occupancy as 10 individual adult offspring produced by a parent plant belonging to a species with a much smaller MAX (Figure 3b). Total mass per individual plant (indicated by the amount of green space) is of course greater for the larger plant in Figure 3a, but there are more canopy mass “gaps” here (represented by the amount of white space) within the perimeter of space occupancy. This pattern, which is apparent here for herbaceous forbs, may be even more pronounced for graminoids, which frequently exhibit a linear growth form that allows high plant densities.

Importantly, if a larger species has biomass that is less densely distributed, the gaps within its occupied space may be available for successful establishment of reproductive individuals belonging only to smaller species, because they have a generally smaller minimum reproductive threshold size (MIN) (Schamp & Aarssen, 2010; Tracey & Aarssen, 2014). This may help account for why relatively small species vastly outnumber larger species (Aarssen et al., 2006). In other words, because a larger species is generally less efficient in harvesting the available resources within its perimeter of space occupancy, it leaves vacant “physical space niches” containing resources that can be harvested and used for reproduction only by smaller species.

Fewer canopy mass gaps in smaller plants (Figure 3B), we suggest, can be expected at least partially as a consequence of the generally higher leafing intensity (number of leaves per unit dry mass of supporting vegetative tissue) in smaller species (Whitman & Aarssen, 2010), and also for smaller plants within species (Scott & Aarssen, 2012). This in turn provides a generally larger bud bank (number of axillary meristems per unit dry mass of supporting vegetative tissue) available for deployment as branches, and hence capacity for a finer scale distribution of dry mass per unit space occupancy. From this then, we would generally expect a 10‐fold decrease in space occupancy per plant (e.g., going from Figure 3a to Figure 3b) to be accompanied by a <10‐fold decrease in dry mass per plant, and this is exactly what our data show, both at the among‐species (Figures 1b,c,e, 2008e) and within‐species (Table 1) level.

We propose that BMD has important implications for predicting plant fitness under crowded conditions. We did not collect fecundity data for our study material. However, previous studies of herbaceous monocarpic species have shown that there is an isometric relationship between fecundity and above‐ground vegetative dry mass—both between species (Aarssen & Jordan, 2001) and within species (Chambers & Aarssen, 2009); that is, fecundity per plant per growing season decreases proportionately with decreasing amounts of vegetative mass produced in the same growing season. Accordingly, while fecundity *per plant* will of course generally be higher for a larger plant (e.g., Fig 3a), we would expect fecundity *per unit mass* to be the same in Figure 3a,b—represented by the density of red circles per unit green space. However, because mass per unit space occupancy is greater for the smaller plant, fecundity *per unit space occupancy* may be higher for the smaller plant—represented by the greater total number of red circles in Figure 3b. This is consistent with findings that indicate that small‐seeded species, which are generally produced by smaller plants, produce more seeds per square meter (Moles, Falster, Leishman, & Westoby, 2004). Thus, we predict that (1) parent plant B (belonging to the smaller species), despite producing fewer offspring, should end up having more grand‐offspring *per unit space occupancy* than parent plant A (belonging to the larger species); and (2) several small crowded plants should collectively produce more offspring than one large plant of the same species occupying the same amount of neighborhood space.

Our results have important implications for the interpretation of “reproductive economy” in plants, that is, capacity to produce at least some offspring despite growth or body size limitation (e.g., due to crowding/competition, or because of limited time available for growth, flowering, pollination, or fruit/seed maturation), and hence the probability of reproducing before death (Aarssen, 2008, 2015; Aarssen et al., 2006). Previous studies indicate that reproductive economy is generally greater in smaller species because of a relatively small minimum reproductive threshold size (MIN) (Tracey & Aarssen, 2011, 2014; Nishizawa & Aarssen 2014). The present results suggest that greater reproductive economy in smaller species may also be promoted by a relatively high fecundity per unit plant body space occupancy—because of a generally higher BMD in smaller species. Future studies that include fecundity data are needed to test this hypothesis more directly.

## Data Accessibility

Data are available from the Dryad Digital Repository: http://dx.doi.org/10.5061/dryad.n6c67 (Tracey et al.^2016^).

## Conflict of Interest

None declared.
