# Supplementary material for: What does body size mean, from the “plant's eye view”?
Source: Ecol Evol. 2016 Sep 22;6(20):7344–51. doi: 10.1002/ece3.2476 (PMC5513261; doi:10.1002/ece3.2476)
Supplement: Supplementary file 1 [file ECE3-6-7344-s001.docx]

# SUPPORTING INFORMATION

**Table S1:** List of the 138 species collected. Bolded are the 10 species that were selected for within-species analyses. Nomenclature follows Gleason and Cronquist (1991).

| Family | Scientific Name | Life History |
| --- | --- | --- |
| Apiaceae | *Daucus carota* L. | biennial |
| Apocynaceae | *Apocynum androsaemifolium* L. | perennial |
| Asclepiadaceae | *Asclepias incarnata* L. | perennial |
|  | *Asclepias syriaca* L. | perennial |
|  | *Vincetoxicum rossicum* N. Wolf | perennial |
| Asteraceae | *Achillea millefollium* L. | perennial |
|  | *Ambrosia artemisiifolia* L. | annual |
|  | *Ambrosia trifida* L. | annual |
|  | *Anaphalis margaritacea* (L.) Benth & Hook | perennial |
|  | *Antennaria neglecta* Greene. | perennial |
|  | *Arctium minus* Schk. | Biennial/perennial |
|  | *Aster cordifolius* L. | perennial |
|  | *Aster lanceolatus* Willd. | perennial |
|  | *Aster novae-angliae* L. | perennial |
|  | *Aster umbellata* Mill. | perennial |
|  | *Carduus acanthoides* L. | biennial |
|  | *Centaurea jacea* L. | perennial |
|  | *Chrysanthemum leucanthemum* L. | perennial |
|  | *Cichorium intybus* L. | perennial |
|  | *Cirsium muticum* (L.) Scop. | perennial |
|  | *Cirsium vulgare* (Savi) Tenore. | biennial |
|  | *Conyza canadensis* (L.) Cronq. | annual |
|  | *Erigeron philadelphicus* L. | biennial |
|  | ***Erigeron strigosus* Muhl. Ex Wild.** | annual |
|  | *Eupatorium maculatum* L. | perennial |
|  | *Euthamia graminifolia* (L.) Nutt. | perennial |
|  | *Helianthus divaricatus* L. | perennial |
|  | *Hieracium aurantiacum* L. | perennial |
|  | *Hieracium kalmii* L. | perennial |
|  | *Hieracium piloselloides* Vill. | perennial |
|  | *Hieracium spp.* | perennial |
|  | *Inula helenium* L. | perennial |
|  | *Lactuca saligna* L. | annual |
|  | *Lactuca serriola* L. | biennial |

| Family | Scientific Name | Life History |
| --- | --- | --- |
| Asteraceae | *Lapsana communis* L. | annual |
|  | ***Matricaria matricarioides* (Less.) Porter.** | annual |
|  | *Matricaria recutita* L. | annual |
|  | *Solidago caesia* L. | perennial |
|  | *Solidago Canadensis* L. | perennial |
|  | *Solidago flexicaulis* L. | perennial |
|  | *Solidago juncea* Aiton. | perennial |
|  | *Sonchus arvensis* L. | perennial |
|  | *Sonchus oleraceus* L. | annual |
|  | *Taraxacum officinale* Weber. | perennial |
|  | *Tragopogon pratensis* L. | biennial |
|  | *Tussilago farfara* L. | perennial |
| Balsaminaceae | *Impatiens capensis* Meerb. | annual |
| Berberidaceae | *Podophyllum peltatum* L. | perennial |
| Boraginaceae | *Cynoglossum officinale* L. | biennial |
|  | ***Echium vulgare* L.** | biennial |
|  | *Hackelia virginiana* (L.) Johnst. | biennial |
|  | *Symphytum* spp. | perennial |
| Brassicaceae | *Alliaria petiolata* (Bieb.) Cavara & Grande | biennial |
|  | *Arabis glabra* (L.) Bernh. | biennial |
|  | ***Barbarea vulgaris* R. Br.** | biennial |
|  | *Brassica rapa* L. | annual |
|  | ***Hesperis matronalis* L.** | biennial |
|  | *Thlaspi arvense* L. | annual |
| Campanulaceae | *Campanula rapunculoides* L. | perennial |
|  | *Campanula rotundifolia* L. | perennial |
|  | *Lobelia cardinalis* L. | perennial |
| Caryophllaceae | *Arenaria serpyllifolia* L. | annual/perennial |
|  | *Cerastium arvense* L. | perennial |
|  | *Cerastium vulgatum* L. | perennial |
|  | ***Dianthus armeria* L.** | annual/biennial |
|  | *Saponaria officinalis* L. | perennial |
|  | *Silene nivea* (Nutt.) Otth. | annual |
|  | *Silene vulgaris* (Moench) Garcke. | perennial |
|  | *Stellaria media* (L.) Villars. | annual |
| Chenopodiaceae | *Chenopodium capitatum* (L.) Aschers. | annual |
| Clusiaceae | *Hypericum perforatum* L. | perennial |
| Convolvulaceae | *Convolvulus arvensis* L. | perennial |
| Crassulaceae | *Sedum acre* L. | perennial/biennial |

| Family | Scientific Name | Life History |
| --- | --- | --- |
| Euphorbiaceae | *Euphorbia cypariassias* L. | perennial |
| Fabaceae | *Coronilla varia* L. | perennial |
|  | *Desmodium spp.* | perennial |
|  | *Lathyrus latifolius* L. | perennial/annual |
|  | *Lotus corniculatus* L. | perennial |
|  | *Medicago lupulina* L. | annual |
|  | *Medicago sativa* L. | perennial |
|  | *Melilotus alba* Medikus. | biennial |
|  | *Melilotus officinalis* (L.) Pallas. | biennial |
|  | *Trifolium dubium* Sibth. | annual |
|  | *Trifolium hybridum* L. | perennial |
|  | *Trifolium pratense* L. | perennial |
|  | *Trifolium repens* L | perennial |
|  | *Vicia cracca* L. | perennial/biennial |
| Geraniaceae | *Geranium maculatum* L. | annual |
|  | *Geranium robertanium* L. | annual |
| Hydrophyllaceae | *Hydrophyllum virginianum* L. | perennial |
| Iridaceae | *Sisyrinchium montanum* Greene. | perennial |
| Labiatae | *Glechoma hederacea* L. | perennial |
|  | *Clinopodium vulgaris* L. | perennial |
| Lamiaceae | *Leonurus cardiaca* L. | perennial |
|  | *Mentha arvensis* L. | perennial |
|  | *Prunella vulgaris* L. | perennial |
|  | *Thymus serpyllum* L. | perennial |
| Liliaceae | *Allium cernuum* Roth. | perennial |
|  | *Smilacina racemosa* (L.) Desf. | perennial |
|  | *Trillium spp.* | perennial |
| Lythraceae | *Lythrum salicaria* L. | perennial |
| Malvaceae | *Malva neglecta* Wallr. | annual |
| Nyctaginaceae | *Mirabilis spp.* | perennial |
| Onagraceae | *Epilobium hirsutum* L. | perennial |
|  | *Oenothera biennis* L. | biennial |
|  | *Oenothera perennis* L. | perennial |
| Orchidaceae | *Epipactis helleborine* (L.) Crantz. | perennial |
| Oxalidaceae | ***Oxalis corniculata* L.** | perennial |
| Plantaginaceae | ***Plantago lanceolata* L.** | perennial |
|  | *Plantago major* L. | perennial |
| Polemoniaceae | *Phlox paniculata* L. | perennial |

| Family | Scientific Name | Life History |
| --- | --- | --- |
| Polygonaceae | *Polygonum persicaria* L. | annual |
|  | ***Rumex acetosella* L.** | perennial |
|  | *Rumex crispus* L. | perennial |
| Primulaceae | *Lysimachia ciliata* L. | perennial |
| Ranunculaceae | *Actaea alba* (L.) Mill. | perennial |
|  | *Anemone canadensis* L. | perennial |
|  | *Anemone virginiana* L. | perennial |
|  | *Aquilegia canadensis* L. | perennial |
|  | *Ranunculus acris* L. | perennial |
| Rosaceae | *Agrimonia gryposepala* Wallr. | perennial |
|  | *Fragaria virginiana* L. | perennial |
|  | *Geum canadense* Jacq. | perennial |
|  | *Geum triflorum* Pursh. | perennial |
|  | *Potentilla argentea* L. | perennial |
|  | *Potentilla recta* L. | perennial |
| Rubiaceae | *Galium mollugo* L. | perennial |
| Scrophulariaceae | *Linaria vulgaris* Miller. | perennial |
|  | *Penstemon hirsutus* (L.) Willd. | perennial |
|  | ***Verbascum thapsus* L.** | biennial |
|  | *Veronica arvensis* L. | annual |
|  | *Veronica peregrina* L. | annual |
|  | *Veronica serpyllifolia* L. | perennial |
| Urticaceae | *Urtica chamaedryoides* Pursh. | perennial |
|  | *Urtica dioica* L. | perennial |
| Verbenaceae | *Phryma leptostachya* L. | perennial |
|  | *Verbena hastata* L. | perennial |
|  | *Verbena simplex* Lehm. | perennial |
|  |  |  |
|  |  |  |
|  |  |  |
|  |  |  |
|  |  |  |

**(a)**

Figure S1(a)

**(b)**

Figure S1(b)

**Fig. S1**. Phylogenetic tree created following Phylomatic (version 3.0, http://www.phylodiversity.net; Webb et al. 2011) for: (a) 97 species from 37 families (not including Asteraceae); and (b) 41 species from the Asteraceae family. Trees were plotted using R (R Development Core Team 2012) and the ape package version 3.4 (Paradis et al. 2004).

Figure S2

**Fig. S2.** Within-species (n=20) relationships for body size metrics involving: *Erigeron strigosus* (a-e); and *Hesperis matronalis* (f-j). The coefficient of determination (r^2^) and the associated P values are from Type I linear regression analysis. Solid lines are from RMA regression analyses; m = RMA slope; t and associated P values test for deviation from the null hypothesis of isometry. Dashed lines are shown only to illustrate the slope angles (not the actual line positions) for hypothetical isometric relationships — 1:1 (in a, b, f, g) where both metrics scale in three dimensions; 3:2 (in c, h) where one metric scales in three dimensions and the other scales in two dimensions, and 3:1 (in d, e, i, j) where one metric scales in three dimensions and the other scales in one dimension.

Figure S3

**Fig. S3.** Within-species (n=20) relationships for body size metrics involving: *Dianthus armeria* (a-e); and *Plantago lanceolata* (f-j). r^2^ and the associated P values are from Type I linear regression analysis. Solid lines are from RMA regression analyses; m = RMA slope; t and associated P values test for deviation from the null hypothesis of isometry. Dashed lines are shown only to illustrate the slope angles (not the actual line positions) for hypothetical isometric relationships — 1:1 (in a, b, f, g) where both metrics scale in three dimensions; 3:2 (in c, h) where one metric scales in three dimensions and the other scales in two dimensions, and 3:1 (in d, e, i, j) where one metric scales in three dimensions and the other scales in one dimension.

Figure S4

**Fig. S4.** Within-species (n=20) relationships for body size metrics involving: *Verbascum thapsus* (a-e); and *Matricaria discoidea* (f-j). r^2^ and the associated P values are from Type I linear regression analysis. Solid lines are from RMA regression analyses; m = RMA slope; t and associated P values test for deviation from the null hypothesis of isometry. Dashed lines are shown only to illustrate the slope angles (not the actual line positions) for hypothetical isometric relationships — 1:1 (in a, b, f, g) where both metrics scale in three dimensions; 3:2 (in c, h) where one metric scales in three dimensions and the other scales in two dimensions, and 3:1 (in d, e, i, j) where one metric scales in three dimensions and the other scales in one dimension.

Figure S5

**Fig. S5.** Within-species (n=20) relationships for body size metrics involving: *Rumex acetosella* (a-e); and *Echium vulgare* (f-j). r^2^ and the associated P values are from Type I linear regression analysis. Solid lines are from RMA regression analyses; m = RMA slope; t and associated P values test for deviation from the null hypothesis of isometry. Dashed lines are shown only to illustrate the slope angles (not the actual line positions) for hypothetical isometric relationships — 1:1 (in a, b, f, g) where both metrics scale in three dimensions; 3:2 (in c, h) where one metric scales in three dimensions and the other scales in two dimensions, and 3:1 (in d, e, i, j) where one metric scales in three dimensions and the other scales in one dimension.

Figure S6

**Fig. S6.** Within-species (n=20) relationships for body size metrics involving: *Oxalis corniculata* (a-e); and *Barbarea vulgaris* (f-j). r^2^ and the associated P values are from Type I linear regression analysis. Solid lines are from RMA regression analyses; m = RMA slope; t and associated P values test for deviation from the null hypothesis of isometry. Dashed lines are shown only to illustrate the slope angles (not the actual line positions) for hypothetical isometric relationships — 1:1 (in a, b, f, g) where both metrics scale in three dimensions; 3:2 (in c, h) where one metric scales in three dimensions and the other scales in two dimensions, and 3:1 (in d, e, i, j) where one metric scales in three dimensions and the other scales in one dimension.

Figure S7

# Fig. S7. Between-species (n=146) relationships for above-ground dry mass verses each of five other metrics of body size recorded for individuals sampled from natural populations. For each species, the individual with the largest above-ground dry mass is paired with its fresh mass (a), estimated canopy volume (b), estimated canopy area (c), height (d), and maximum lateral extent (e). r^2^ and the associated P-values are from Type I linear regression analysis. Solid lines are from RMA regression analyses; m = RMA slope; t and associated P values test for deviation from the null hypothesis of isometry. Dashed lines are shown only to illustrate the slope angles (not the actual line positions) for hypothetical isometric relationships — 1:1 (in a, b) where both metrics scale in three dimensions; 3:2 (in c) where one metric scales in three dimensions and the other scales in two dimensions, and 3:1 (in d, e) where one metric scales in three dimensions and the other scales in one dimension.
